# An annotated dataset for identifying behaviour change based on five doors theory under coral bleaching phenomenon on Twitter

**DOI:** 10.1016/j.dib.2021.107617

**Published:** 2021-11-20

**Authors:** Gabriela Nathania Harywanto, Juan Sebastian Veron, Derwin Suhartono

**Affiliations:** Computer Science Department, School of Computer Science, Bina Nusantara University, Jakarta 11480, Indonesia

**Keywords:** Coral bleaching, Five doors theory, Behaviour change, Coral conservation, Automatic classification

## Abstract

Behaviour change is the target ultimate of environmental campaigns that are being intensively carried out by various parties. One of the environmental issues of global concern is coral bleaching. Coral bleaching threatens biodiversity and the balance of ecosystems around the world because marine ecosystems are the foundation of life on this earth [1]. Social media data can be very useful for conservation [2], including in monitoring behaviour changes. The crawling process of data from the Twitter social media platform has been carried out from early 2021 to May 2021 periodically. Obtained 1,222 tweets that have been carefully filtered and labelled into stages of behaviour change by three expert annotators. There are five stages of behaviour change based on the Five Doors Theory: desirability, enabling context, can do, buzz, and invitation [3]. Labelling is done qualitatively and guided by annotation rubrics that have been made based on linguistic patterns at each stage of behaviour change [4]. The data that has been created is expected to be used by various parties working in the field of coral conservation, especially psychologists and data scientists. This data can be used as a basis for analysing behaviour change and used to build an automatic classification model as a means of evaluating and monitoring the behaviour change of Twitter users on the phenomenon of coral bleaching.

## Specifications Table

**Table:** 

Subject	Computer Science Management, Monitoring, Policy and Law Social and Personality Psychology
Specific subject area	Tweet mining for identification of behaviours change on coral bleaching phenomenon
Type of data	Text
How data were acquired	The data was crawled from Twitter API and qualitatively annotated by three experts according to 5 stages of behaviour change on Five Doors Theory [3]
Data format	Raw (primary data) Labelled (secondary data)
Parameters for data collection	Tweets were collected if they contain keywords: “coral bleaching”, “ocean warming”, “coral restoration”, or “coral monitoring”
Description of data collection	The data was crawled with specific keywords as primary data. Crawling process is done periodically, starting from January 2021 to May 2021. For secondary data, all the data are prepared for labelling process, which were standardized with annotation rubric. The data has been filtered for non-English, relevance, and duplication. Two expert annotators were qualitatively and manually labelling the data according to 5 stages of behaviour change. If there was any split decision between two annotators, then the data will be passed into the additional annotator for a majority vote.
Data source location	Twitter
Data accessibility	Repository name: Mendeley Data Data identification number: 10.17632/hfdg5297kc.4 Direct URL to data: https://data.mendeley.com/datasets/hfdg5297kc/4

## Value of the Data

• These data are useful for identifying and monitoring behavioural change stage which help to develop interventions leading to a desired behaviour change under coral bleaching phenomenon topic on Twitter.
• This dataset can be used by everyone who have interest for exploring study of behaviour change toward coral bleaching, such as conservationist, psychologists and also data scientists.
• For larger scale studies, this dataset can be used to analyse the behaviour stage of each Tweet's writer towards coral bleaching by providing the basis for designing and building classification model to automatically classify 5 stages of behaviour changes.
• These data were collected carefully from the beginning of the year of 2021 and periodically crawled dataset, which shows different trend that happens in the world that are related with coral bleaching.

## 1 Data Description

There were 2196 tweets that have been crawled from twitter and then processed to build this dataset. After filtering and annotating process, the dataset contains 1222 rows with 11 columns (*decision, created_at, id, id_str, full_text, source, retweet_count, favorite_count, reply, username_length,* and *user_location*). The definition of each column name can be in Table 1. Each row is a data from single tweet which posted on twitter at the time.

**Table 1:** Columns in the dataset and its descriptions.

Column	Description
*decision*	Classification of behaviour change based on Five Doors Theory. Example: ”desirability”

*created_at*	UTC time when this Tweet was created. Example: “Wed Oct 10 20:19:24 +0000 2018”

*id*	The integer representation of the unique identifier for this Tweet. Example: 1050118621198921728

*id_str*	The string representation of the unique identifier for this Tweet. Example: “1050118621198921728”

*full_text*	The actual UTF-8 text of the status update. Example: ”To make room for more expression, we will now count all emojis as equal—including those with gender‍‍‍ ‍‍and skin t… https://t.co/MkGjXf9aXm”

*source*	Utility used to post the Tweet, as an HTML-formatted string. Tweets from the Twitter website have a source value of web. Example: “Twitter Web Client”

*retweet_count*	Number of times this Tweet has been retweeted. Example: 160

*favorite_count*	Indicates approximately how many times this Tweet has been liked by Twitter users. Example: 295

*reply*	Represents is the Tweet is a replying another user. ‘TRUE’ for replying Tweet and ‘FALSE’ for not. Example: TRUE

*username_length*	The username length of user who posted the Tweet Example: 7

*user_location*	The country-based location of user who posted the Tweet. If there are several countries or no clear and sufficient geographical information, this column will be valued as “Unknown” Example: “Canada”

**Fig. 1:**
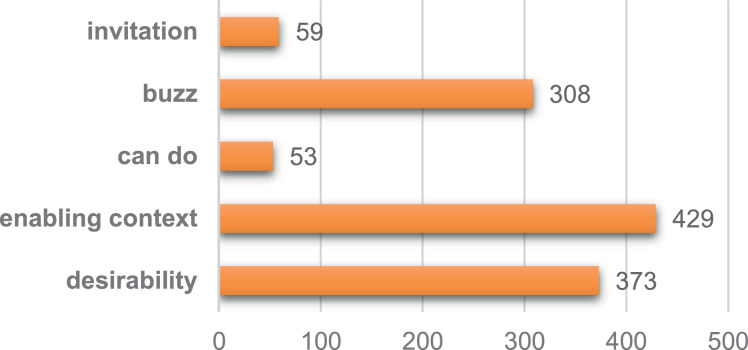
Distribution of each class on *decision* column.

Each tweet has been annotated with one out of 5 stages of behaviour change based on Five Doors Theory [3]. The annotation result can be seen in column *decision*. Five stages of behaviour change are: desirability, enabling context, can do, buzz, and invitation. Every stage has their own characteristic [5]. In desirability stage, someone is motivated to reduce their frustration which can be about everyday inconvenience or about more personal frustration. In enabling context stage, people are changing their surrounding environment to enable new behaviour. In can do stage, people are already acting and focused on self-efficacy and lowering the perceived risks. In buzz stage, people share their experience and success stories to crate buzz and increase other's desires. In invitation stage, people invite and engage others to their cause. Each stage has its own linguistic pattern on social media post [4], shows in Table 2. The distribution of each class on *decision* column can be seen in Fig. 1. There is an imbalanced distribution because the data are collected from actual sources, according to the conditions in the field. In can do and invitation classes only have approximately 9% of the total data portion. While the other 3 classes (desirability, enabling context, and buzz) have a fairly even portion in the range of 20% to 35% of the total data. The imbalance in the distribution of data in each class may occur due to the nature of social media where people are more likely to use social media to share their complaints, thoughts, desires, and success stories. This is in accordance with the characteristics of the 3 dominant classes (desirability, enabling context, and buzz): exposing frustrations, conveying knowledge and suggestions for change, and telling success stories.

**Table 2:** Linguistic patterns per behavioural stage.

Behaviour Stage	Common Linguistic Pattern
Desirability	- Negative sentiment (expressing personal frustration- anger/sadness) - URLs (generally associated with facts) - Questions (how can I? / what should I?)

Enabling Context	- Neutral sentiment - Conditional sentences (if you do [..] then [...]) - Numeric facts [consumption/pollution] + URL.

Can Do	- Neutral sentiment - Orders and suggestions (I/we/you should/must...)

Buzz	- Positive sentiment (happiness/joy) - I/we + (present tense) I am doing/we are doing

Invitation	- Positive sentiment (happy/cute) - [vocative] Friends, guys - Join me/tell us/with me

The full text of the tweet is not subject to pre-processing so that gives freedom to further user of this data. In the actual world, there are a lot of same tweets so that this dataset has been filtered so there are no duplicates. To ensure the privacy of all crawled tweet, the dataset only give the length on of the username of user account which post the tweet. The time when the tweet was posted can be referred on column *created_at* with format of date (ISO 8601). All the columns, except *decision*, are collected and based on JSON file of crawled tweets via Twitter API at the time.

**Fig. 2:**
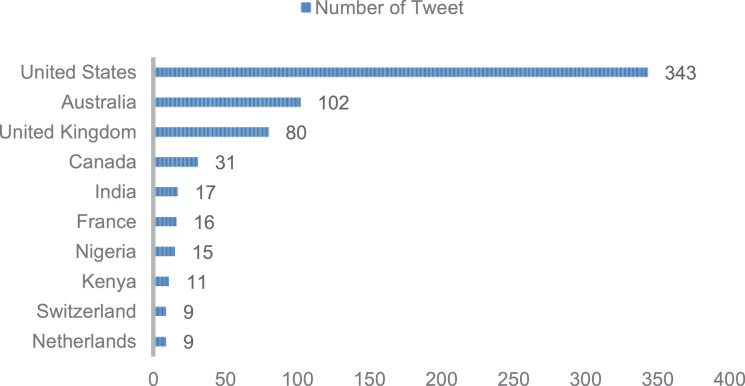
The distribution of user's location based on country.

**Fig. 3:**
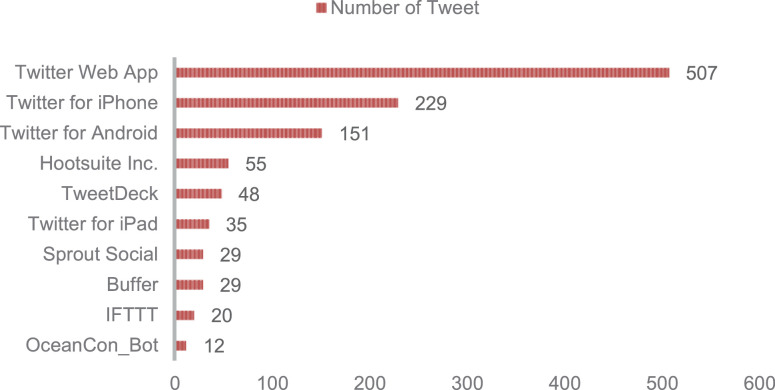
The distribution of utility which user used to send tweet.

Twitter users are spread all over the world. However, it is undeniable that the issue of coral bleaching on Twitter has only become a trend in certain countries. Fig. 2 shows that the United States dominates the origin of users who send tweets, followed by Australia and the United Kingdom. Australia ranks second in line with the country having the longest stretch of coral reef, the Great Barrier Reef. Actually, not all tweets can be identified by the location of the user who sent them. A total of 460 tweets were not identified with certainty user country, due to limited geographic information and unclear information. Fig. 3 shows the distribution of users' use of the utility to send tweets. The use of Twitter Web App is the most popular, followed by Twitter for iPhone, Twitter for Android, and other utilities that are not very popular.

**Fig. 4:**
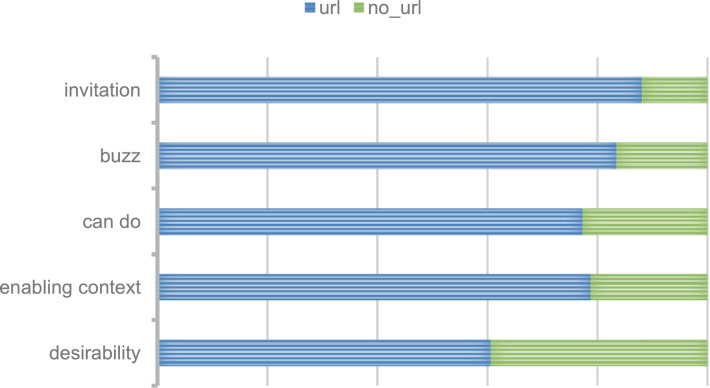
Proportion of *full_text* contain link or not on each class. The blue bars show the *full_text* that contain link and the green bars show the *full_text* without link.

In the *full_text* data there are links that refer to certain web pages or refer to the media in the tweet. Overall, each class has more *full_text* with link than without link (Fig. 4). In desirability and enabling context, the links usually contain facts and media such as supporting photos. In can do and invitation, the link usually refers to a campaign page or registration page to join a program. In buzz, the link usually refers to media in the form of photos or news about success stories.

## 2 Experimental Design, Materials and Methods

The data were crawled using Twitter API with Tweepy library on python. Only tweet containing specific keywords ("coral bleaching", "ocean warming", "coral restoration", and "coral monitoring") would be crawled. All retweeted tweets were eliminated. The crawling process take place periodically starting from January 2021 until May 2021. There were three main session of crawling process which took time around January, March, and May. In each session, data crawling is carried out four times in 4 weeks (once a week). Crawling process was done every week because standard Twitter Search API only can search against a sampling of recent Tweets published in the past 7 days. The crawling process not only gather full text of the tweet, but also other corresponding information of that tweet (e.g., time when posted, user who post the tweet, retweet count, etc). All crawled tweet data was stored in JSON format.

**Fig. 5:**
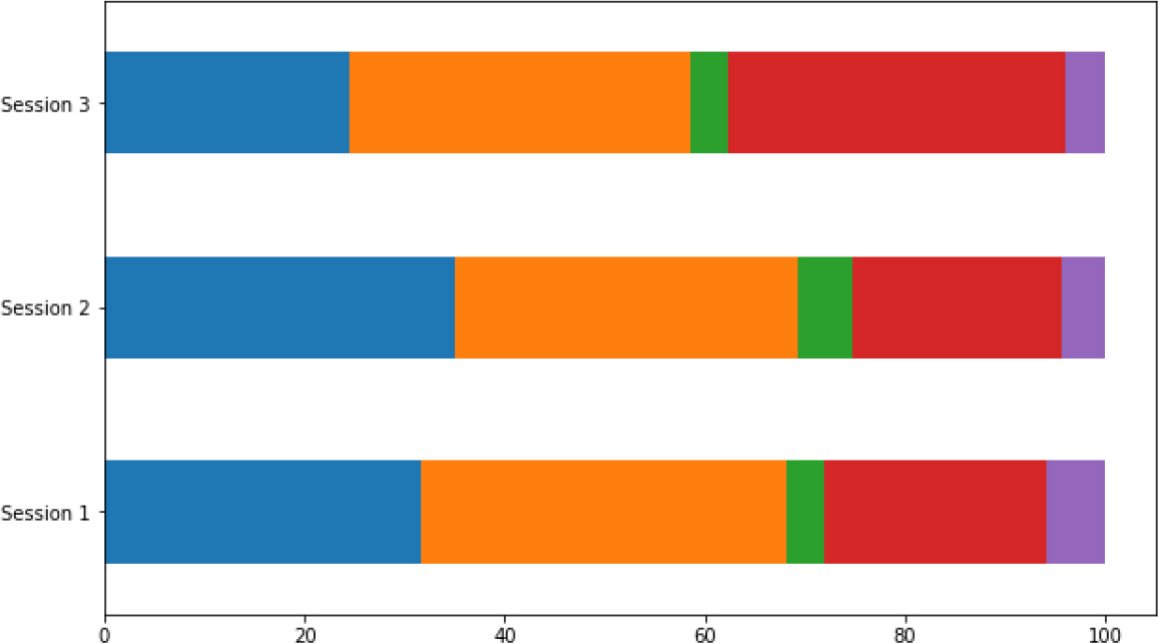
Proportion of each class on every session. Note: blue bars: desirability class, orange bars: enabling context class, green bars: can do class, red bars: buzz class, and purple bars: invitation class.

**Fig. 6:**
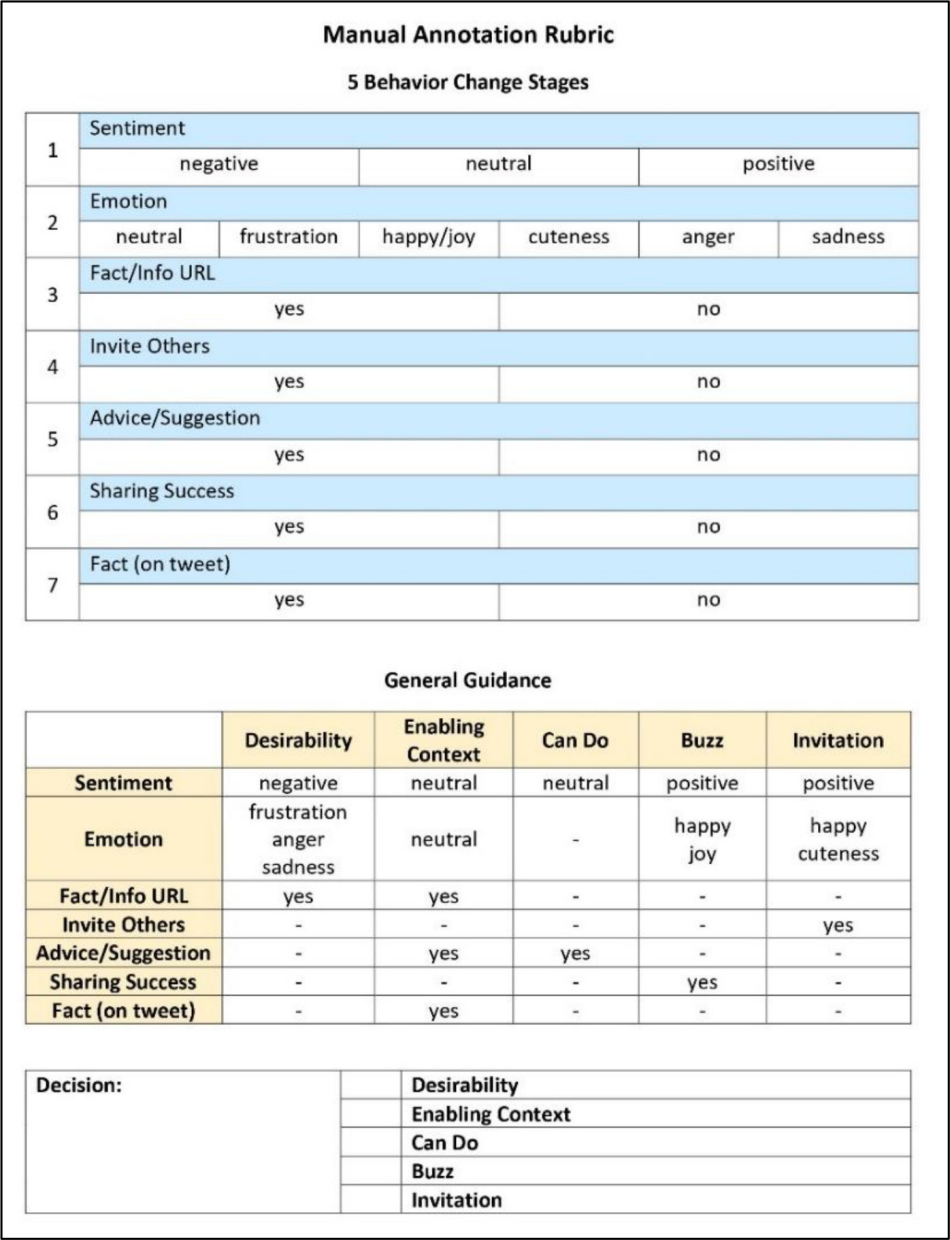
Manual annotation rubric.

After all desired tweets were crawled, this data was prepared to be labelled by 2 expert annotators. Literally, the annotators are people in the field of computer science, but they also have knowledge in coral conservation, so they can be reliable annotators in this case. Only full text of the tweet is used for determining decision on class label. The labelling process is based on linguistic pattern on each class. Simple annotation rubric (Fig. 6) is created to help each annotator for giving a standardized qualitative assessment to label the data. The data also filtered manually by the annotators from non-English, duplication and irrelevance topic or context (such as hate speech, debating argumentation, joke, and irony which just only contains specific keyword for collecting data). The number of duplication and irrelevant tweet were dominated the rejected data. Total number of duplication Tweet is 558 and the total number of irrelevant Tweet is 321, also the non-English Tweet is 95. The number of irrelevant Tweet also cover the duplication of that, whereas the number of duplication Tweet itself only cover the duplication of selected data.

Two expert annotators were labelling the data manually and doing it in multiple session. On every session, they would discus and determine each tweet decision label. If there are unanimous decision (two annotators agreed on a label), the data will be put into the agreed label, but if there are split decision (two annotator contradict on a label), then the data will be passed into the additional annotator for a majority vote. From all the tweets data which labelled by two expert annotators, the Krippendorff's Alpha (α) was calculated to measure the reliability of the data. The α result were 0.928. It shows an acceptable reliability value (above 0.8) for the data created [6].

After the data labelling process is complete, the distribution of class proportions for each data collection session shows that in each session the distribution is always dominated by 3 classes (desirability, enabling context, and buzz). From the visualization in Fig. 5, it can also be seen that there has been no significant change in the trend of the stages of behavioural change. In the third session, it was found that there was a higher percentage of buzz classes than in the first and second session, because at that time there was a new coral restoration program being launched, so there were many posts about it. This study has some limitation: only consisted of 3 sessions of data collecting, accepted only English tweet, and not suggested any further intervention strategy for each stage of behaviour.

## Ethics Statement

All data are fully anonymized and were collected and distributed under Twitter's Developer Policy 2021 [7].

## CRediT Author Statement

**Gabriela Nathania Harywanto:** Conceptualization, Data curation, Writing – original draft; **Juan Sebastian Veron:** Data curation; **Derwin Suhartono:** Supervision.

## Declaration of Competing Interest

The authors declare that they have no known competing financial interests or personal relationships which have or could be perceived to have influenced the work reported in this article.
